# First Cases of Natural Infections with *Borrelia hispanica* in Two Dogs and a Cat from Europe

**DOI:** 10.3390/microorganisms8081251

**Published:** 2020-08-18

**Authors:** Gabriele Margos, Nikola Pantchev, Majda Globokar, Javier Lopez, Jaume Rodon, Leticia Hernandez, Heike Herold, Noelia Salas, Anna Civit, Volker Fingerle

**Affiliations:** 1 German National Reference Centre for Borrelia, Bavarian Health and Food Safety Authority, 85764 Oberschleißheim, Germany; volker.fingerle@lgl.bayern.de; 2 IDEXX Laboratories, 70806 Kornwestheim, Germany; nikola-pantchev@idexx.com (N.P.); majda-globokar@idexx.com (M.G.); 3 IDEXX Laboratories, 08038 Barcelona, Spain; javier-lopez@idexx.com (J.L.); jaume-rodon@idexx.com (J.R.); leticia-hernandez@idexx.com (L.H.); noelia-salas@idexx.com (N.S.); anna-civit@idexx.com (A.C.); 4 Bavarian Health and Food Safety Authority, 85764 Oberschleißheim, Germany; herold.heike@web.de

**Keywords:** *Borrelia hispanica*, Europe, relapsing fever, borreliosis, cat, dog

## Abstract

Canine cases of relapsing fever (RF) borreliosis have been described in Israel and the USA, where two RF species, *Borrelia turicatae* and *Borrelia hermsii*, can cause similar clinical signs to the *Borrelia persica* in dogs and cats reported from Israel, including fever, lethargy, anorexia, thrombocytopenia, and spirochetemia. In this report, we describe the first clinical cases of two dogs and a cat from Spain (Cordoba, Valencia, and Seville) caused by the RF species *Borrelia hispanica.* Spirochetes were present in the blood smears of all three animals, and clinical signs included lethargy, pale mucosa, anorexia, cachexia, or mild abdominal respiration. Laboratory findings, like thrombocytopenia in both dogs, may have been caused by co-infecting pathogens (i.e., *Babesia vogeli*, confirmed in one dog). Anemia was noticed in one of the dogs and in the cat. *Borrelia hispanica* was confirmed as an infecting agent by molecular analysis of the 16S rRNA locus. Molecular analysis of housekeeping genes and phylogenetic analyses, as well as successful in vitro culture of the feline isolate confirmed the causative agent as *B. hispanica*.

## 1. Background

The genus *Borrelia* comprises three phylogenetic clusters, namely: Lyme Borreliosis (LB) borreliae, relapsing fever (RF) borreliae, and the recently described reptile-associated and echidna-associated *Borrelia* (REP borreliae, *Borrelia (B.) turcica,* and *Candidatus* B. tachyglossi, respectively) [1,2].

LB in dogs in Europe has been described as a condition of arthritis and/or glomerulonephritis; the incidence of clinical disease is rare, despite a high exposure of dogs to the tick vector (*Ixodes ricinus*), and there may be a breed/genetic predisposition (reviewed by the authors of [3]). LB in cats in Europe is a rare condition—the seroprevalence is significantly lower compared with dogs, which may, in part, be explained by different tick exposures [4].

Apart from LB species, which are vectored by hard ticks of the genus *Ixodes*, soft ticks in Europe may carry *Borrelia* species that belong to the RF group of spirochetes, in particular *Borrelia hispanica*. *Borrelia hispanica* represents a spirochete species transmitted by *Ornithodoros* ticks, which can cause TBRF (tick-borne relapsing fever) in humans in the Mediterranean area [5,6]. Trape et al. [7] detected *B. hispanica* in *O. marocanus, O. occidentalis,* and *O. kairouanensis*. They represent a complex of three large species of soft ticks (Argasidae) of the genus *Ornithodoros* (female adult length 5.5–7.5 mm), and are distributed in characteristic Mediterranean regions in Morocco, Algeria, Tunisia, and Spain. These species were previously confused with *O. erraticus* [7]. Up until now, however, no cases of *B. hispanica* in dogs and cats have been reported in Europe.

Cases of RF borreliosis have been reported in dogs and cats from Israel naturally infected with *B. persica*, a *Borrelia* species that is transmitted by *O. tholozani* in the Middle East, Egypt, Central Asia, and India. The main clinical findings in cats included lethargy, anorexia, anemia, and thrombocytopenia, while fever (>39 °C) was reported in only one out of five cats. Dogs were lethargic, anorexic, anemic, and showed fever; the majority were also thrombocytopenic. Three dogs out of five were co-infected with *B. persica* and *Babesia*. The animals were all treated with antibiotics (tetracyclines, penicillins, fluorochinolons, or combinations of them), the dogs co-infected with *Babesia* received additional imidocarb diproprionate, and the survival rate of both dogs and cats was 80% [8].

Furthermore, canine cases of RF borrelia are described from the U.S., where two RF species, *B. turicatae* and *B. hermsii*, transmitted by *Ornithodoros* ticks, can cause similar clinical signs as *B. persica* in dogs and cats, including fever, lethargy, anorexia, thrombocytopenia, and spirochetemia. Neurologic disease symptoms have also been described [9].

In this report, we describe the first clinical cases of two dogs and a cat caused by *B. hispanica* in Europe, with spirochetes present in blood smears, molecular analysis, and successful in vitro cultivation of one isolate.

## 2. Case Reports

### 2.1. Canine Case 1 (Dog 1; Sample ID VM531519)

Anamnesis: Podenco (hound); short hair; male; 6 years old; approximately 10 kg body weight (BW); hunting dog living in La Luisiana, Seville (Spain); and sleeps under a roof. The following prophylactic measures were established: imidacloprid, chlorfenvinphos, and diazinon; no prophylaxis for leishmaniosis; treatment for internal parasites using albendazole and a combination of praziquantel/pyrantel/febantel; and annual rabies vaccine plan.

**Figure 1:**
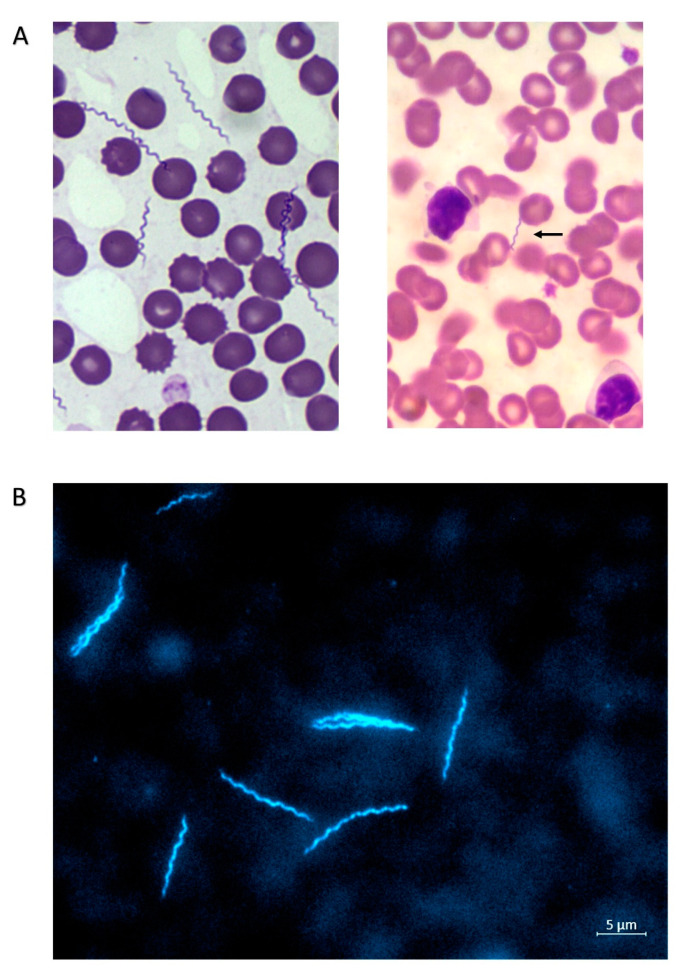
Spirochetes in Giemsa-stained blood smears of dog 1 (**A**, left panel, magnification 150×), dog 2 (**A**, right panel, magnification 100×, arrow pointing to *Borrelia*), and 4′,6-diamidino-2-phenylindole (DAPI) stained *Borrelia* from an in vitro culture of infected cat blood (**B**).

The dog was presented to a local veterinarian in February (3 February 2014) being lethargic, with pale mucosa, anemia (hematocrit 30.2%), and thrombocytopenia (Table 1). No data about the occurrence of microorganisms in a stained blood smear were available at this time point. Because of a positive *Ehrlichia canis* serologic result (SNAP^®^ 4Dx^®^), treatment was performed with 5 mg/kg/BW of doxycycline, every 12 h, for three weeks. Despite a good appetite, at the end of the first doxycycline cycle (27 February 2014), the hematocrit was still low (20.9%). In March (4 and 13 March 2014), the hematocrit was still not within the reference range (38.3–56.5%), with values of 32.8% and 31.4%, respectively. Therefore, a second course of doxycycline, as described above, was performed. The dog improved clinically and by the end of March 2014 showed no more anemia. On 30 October 2014, the dog showed non-regenerative anemia (hematocrit 29.4%, erythrocytes 3.98 m/µL, hemoglobin 8.9 g/dL, and reticulocytes 58,108/µL), thrombocytopenia (23 tsd.), mild lymphopenia, and eosinopenia, as well as many spirochetes in a Giemsa-stained blood smear (Figure 1A, left panel). A diagnosis of relapsing fever was established and treatment with doxycycline was started the following day at the dose and interval mentioned above. Unfortunately, no information is available about the efficacy of the treatment and clinical progress.

The laboratory tests were performed at IDEXX Ludwigsburg, Germany (November 2014).

PCR: At IDEXX Ludwigsburg, a diagnostic real-time PCR was conducted for *B. burgdorferi* sensu lato (target gene was flagellin B (*flaB*)) with a negative result.

Serology: the dog showed 66 U/mL in the Quant-C6-Enzyme-linked immunosorbent assay (ELISA) (IDEXX; cut off 10 U/mL), 25 VE (Virotech units) measured by a whole-cell based IgG ELISA (Virotech; without VlsE; cut off 12 VE). The SNAP^®^ 4Dx^®^ (IDEXX) test was negative, apart from a positive reaction against p30/p30-1 peptides of *Ehrlichia canis*. The *Borrelia* immunoblot (Viramed) showed two positive bands (p14 and VlsE), and four weakly positive bands (DbpA (Osp17), p21, OspC, and p39; data not shown). The sample showed a very high *E. canis* antibody titre in the immunofluorescence assay (IFA; MegaCor; >1:2560 cut off 1:40), a low *Leishmania* antibody level in ELISA (Afosa; 15.8 test units (TU); positive cut off 12 TU), and also a low positive *Babesia* antibody level of 23.5 TU (Afosa; positive cut off 19 TU).

### 2.2. Canine Case 2 (Dog 2; Sample ID VM736940)

Anamnesis: This dog lives in the Gandia area (Valencia), in a residential area in the countryside. It is an outdoor dog and sleeps in the courtyard. It was usually dewormed with a combination of febantel/pyrantel/praziquantel, and treated for ectoparasites with a deltamethrin-containing collar on an irregular basis. The dog showed anorexia and apathy at a body temperature of 39 °C (Table 1). Within a blood smear, low level of *Babesia* spp. was observed and only few *Borrelia* were visible (Figure 1A, right panel); the dog showed no anemia, but only leukocytosis (20.3 g/L) based on lymphocytosis (11,182/µL) and monocytosis (1130/µL), as well as thrombocytopenia (92 tsd.). Further blood values (including clinical chemistry) were within the normal range.

Serology (IDEXX Ludwigsburg): The SNAP^®^ 4Dx^®^ (IDEXX) test as well as two *Borrelia* ELISA tests, Quant-C6-ELISA (IDEXX) and the whole-cell based IgG ELISA (Virotech^®^), were negative (no immunoblot was performed).

PCR (IDEXX Ludwigsburg): Real-time PCRs performed for *Leishmania* spp. (GP63), *Ehrlichia* spp. (*dsb*), *Leptospira* spp. (*lipl32/hap-1*), *B. burgdorferi* sensu lato (*flaB*), *Anaplasma platys* (*groEL*), and *Anaplasma phagocytophilum* (*msp2*) were negative; *Babesia vogeli* DNA (hsp 70) was amplified.

Treatment with doxycycline (for four weeks) and amoxicillin (for two weeks) was started after the suspected diagnosis of a spirochetal infection. After treatment, the dog recovered completely.

### 2.3. Feline Case (Cat; Sample ID 10827448)

Anamnesis: one-year old street cat, male, plenty of fleas, and no tick prophylaxis.

At presentation, the cat showed cachexia (extreme weight loss), mild abdominal respiration, and had a good appetite (Table 1). The body temperature was not elevated at the time of *Borrelia* detection. The cat was living in the Cordoba area (Andalucia). Laboratory abnormalities included severe regenerative anemia (low erythrocytes (2.45 m/µL), hemoglobin (3.3 g/dL), and hematocrit (12.9%) at high reticulocyte numbers (139,895/µL)) and mild monocytosis. No other clinic-pathological abnormalities were found.

Serology (IDEXX Ludwigsburg): FeLV (Feline leukemia virus antigen)/FIV (feline immunodeficiency virus antibodies) (Uranotest^®^) and SNAP^®^ 4Dx^®^ (IDEXX) tests were performed and came back negative; spirochetes were clearly visible in a Giemsa-stained blood smear (not shown).

Treatment was started with doxycycline after an additional blood sample was drawn for culture and molecular analyses. The duration of antibiotic treatment was 30 days. No clinical signs were observed after the treatment, and all of the altered values of hematology returned to normal.

## 3. Methods

### 3.1. Samples Included in Our Study

All of the samples originated from animals living in Spain. The samples were sent to the IDEXX laboratory in Barcelona from local veterinarians, where the animals were presented because of clinical signs. The samples were obtained as part of a routine diagnostic evaluation. Written informed consent was obtained from the owner. All investigations comply with the current laws of the countries in which they were performed. Diagnostic laboratory analyses (serology and PCR) were performed at the IDEXX reference laboratories in Ludwigsburg/Germany, and were subsequently sent for culture and further molecular analyses to the German National Reference Center for Borrelia at the Bavarian Health and Food Safety Authority, Oberschleißheim/Germany. The sample IDs are as follows: dog 1 = VM531519; cat = 10827448; dog 2 = VM736940.

### 3.2. Serology IDEXX Ludwigsburg, Germany

All of the tests were performed according to the literature [3,10,11]. For the immunoblot, slight modifications were implemented. Briefly, the Borrelia + OspA/B ViraStripe IgG Testkit (Viramed; Planegg, Germany) was performed, which contains specific purified antigens from *B. afzelii* (PKo) and *B. burgdorferi* sensu stricto, as well as a recombinant VlsE. As a secondary antibody, a phosphatase-labeled affinity purified antibody to dog IgG (H+L; produced in goat) at a dilution of 1:500 was used.

### 3.3. PCRs IDEXX Ludwigsburg, Germany

All of the tests were performed according to the literature [10,11], apart from *Borrelia* and *Leptospira*. Briefly, the total nucleic acid was extracted from the blood by using a QIAamp DNA Blood Mini kit (QIAGEN; Hilden, Germany), according to the manufacturer’s instructions. A real-time-PCR assay was performed using the LightCycler 480 (Roche, Mannheim, Germany) with proprietary forward, reverse primers, and hydrolysis probes. The target gene for *Leptospira* spp. was the *lipl32/hap-1* (accession number AF245281.1) and for *B. burgdorferi* sensu lato *flaB* (MF150071.1). The PCR assays that were employed during this study were shown to have a reproducible average analytical sensitivity of 10 DNA molecules per reaction.

### 3.4. In Vitro Culture from Cat Blood at NRZ Borrelia, Oberschleißheim, Germany

The Ethylenediaminetetraacetic acid (EDTA) blood obtained from the cat was used to set up in vitro cultures in microwell plates. All of the cultures were kept at 33 °C under a 5% CO_2_ atmosphere. Four different media were used for the in vitro cultivation: (1) Modified-Kelly-Pettenkofer (MKP) medium (MKP basic medium supplemented with 5% bovine serum albumin and 6% rabbit serum) [12], (2) Barbour-Stoenner-Kelly (BSK)-H complete medium (Sigma-Aldrich; Darmstadt, Germany), (3) BSK-Y medium, and (4) RF-medium (MKP basic medium supplemented with bovine serum albumin (5%) and 50% fetal calf serum (FCS); CC-pro, Germany) [13]. For all of the cultures, 10 μL of EDTA blood was placed into 300 μL of medium.

### 3.5. DNA Extraction and PCR (NRZ Borrelia, Oberschleißheim)

DNA was extracted from the EDTA blood using the Maxwell^®^ 16 Blood DNA Purification Kit according to the manufacturer’s instructions (Promega, Mannheim, Germany). The extracted DNA was subjected to PCR amplification, as described below.

Fragments of the 16S rRNA were amplified using primers and PCR conditions, as described previously [14]. Multilocus sequence typing (MLST) on housekeeping genes (*clpA, clpX, nifS, pepX, pyrG, rplB, recG,* and *uvrA*) was performed principally, as described (see www.pubmlst.org/borrelia, [15]). The sequences for the primers used are given in Table 2. For all of the PCR reactions, HotStarTaq Mastermix (Qiagen, Germany) was used. A touch-down protocol was employed for the first nine cycles with annealing temperatures of 55 °C to 48 °C, decreasing by 1 °C each cycle, followed by 32 cycles at a 48 °C annealing temperature. The temperature profile was 95 °C for 15 min (activation of Taq polymerase), denaturation 94 °C for 30 s, annealing 30 s, and elongation 72 °C for 60 s. A final step of elongation was at 72 °C for 5 min, and the samples were maintained at 12 °C.

### 3.6. Molecular Analyses

Commercial sequencing was done by GATC Biotech AG (Konstanz, Germany). We used MEGA5 [16,17] for sequence alignment, genetic distance analyses, and construction of phylogenetic trees. The Basic Local Alignment Search Tool (BLAST) [18] was used to compare the sequences obtained here to the sequences in GenBank using standard settings. Genetic distance analyses were conducted in MEGA5 [16,17] using the Kimura 2-parameter model [19]. The evolutionary history was inferred by using the maximum likelihood method based on the general time reversible model [16,20]. The initial tree(s) for the heuristic search were obtained automatically by applying neighbor-joining and BioNJ algorithms to a matrix of pairwise distances estimated using the maximum composite likelihood (MCL) approach, and then selecting the topology with a superior log likelihood value. The bootstrap values were calculated for 1000 repetitions. A discrete Gamma distribution was used to model the evolutionary rate differences among sites (+G). The rate variation model allowed for some sites to be evolutionarily invariable (+I). The trees are drawn to scale, with branch lengths measured in the number of substitutions per site. The codon positions included were 1st+2nd+3rd+Noncoding for the housekeeping gene sequences. All of the positions containing gaps and missing data were eliminated. Further information is given in the figure legend.

### 3.7. Sequence Deposition

The 16S rRNA sequences were submitted to GenBank with accession numbers MN173954 (cat) and MN175320 (dog2). The sequences of the MLST housekeeping loci can be obtained from the *Borrelia* MLST database under pubmlst.org/borrelia/. Allele numbers are given in Table 3.

## 4. Results and Discussion

The report presented here describes the first clinical cases of natural infections with *B. hispanica* in two dogs and one cat living in various places in Spain (Cordoba, Valencia, and Seville). Clinical signs were reported as lethargy, pale mucosa, anorexia, cachexia, or mild abdominal respiration, and were consistent with previously described symptoms in animals infected with different RF *Borrelia* species [8,9].

Dog 1 (sample ID VM531519) represents a case with clearly visible spirochetes in the blood (Figure 1A) that were identified as *B. hispanica* by molecular analysis. This dog had the following antibodies to other canine vector-borne diseases (CVBDs): high *E. canis* titer but also low *Leishmania* and *Babesia* antibodies. Thus, the clinical signs and laboratory abnormalities (such as non-regenerative anemia and thrombocytopenia, as well as a mild lymphopenia and eosinopenia) could have been triggered or exacerbated by the presence of a co-infection. Another explanation may be that the dog experienced an *Ehrlichia*-infection in early February 2014—which was successfully treated—and then acquired an RF *Borrelia* infection that was diagnosed in October 2014. Co-infections in animals with different disease agents should always be considered; in our study, dog 2 (sample ID VM736940) showed a co-infection of *B. hispanica* (microscopically/molecular) and *Ba. vogeli* (microscopically/molecular). In a previous study on dogs infected with *B. persica* and co-infected with *Babesia* (PCR and microscopy/blood smear), additional treatment with imidocarb was initiated [8], but this was not performed in the present case as dog 2 improved with only antibiotic treatment (amoxicillin and doxycycline).

Interestingly, dog 1 showed a positive serologic reaction to *B. burgdorferi* in ELISA, whereas dog 2 did not. Possible reasons for these differences in the diagnostic response include an actual exposure to both (relapsing fever and Lyme borrelia) in dog 1; an anamnestic titer against *B. burgdorferi*, after a previous successfully cleared infection; or a varied cross-reactivity of antibodies (i.e., IgM) against RF *Borrelia* with *B. burgdorferi* due to different time points of infection. Support for the latter supposition comes from the fact that dog 1 showed a high number of spirochetes in the blood smear (Figure 1A, left panel), whereas only a few bacteria were identified in the blood smear of dog 2 (Figure 1A, right panel). Moreover, dog 2 was the only animal without anemia, and showed signs pointing toward chronic infection, such as leucocytosis and thrombocytopenia. A recent experimental infection of six dogs with the RF species *B. turicatae* lends support to the cross-reactivity hypothesis, as five of the dogs showed positive results in a whole-cell-based test (IFA) for LB borreliae, three of them had slight positive reactions in a Quant-C6-ELISA (13 to 24 U/mL), but all six dogs reacted negatively in the SNAP^®^ 4Dx^®^ test (as shown for both dogs and the cat in the present study) [21]. A limitation of this study was that only one time point post infection (43 dpi) was tested. Therefore, the SNAP^®^ 4Dx^®^ test utilizing a C6 peptide of *B. burgdorferi* can currently be regarded as the only serological test for LB in dogs not cross-reacting with RF borreliae. At present, there are no commercially available serological tests for RF borreliosis in dogs that could improve the diagnosis, i.e., based on GlpQ and BipA [21]. Another RF *Borrelia* species in Europe, *B. miyamotoi*, may represent also a diagnostic challenge in terms of cross-reactivity with LB borreliae. Because it has the same vector as *B. burgdorferi,* co-exposure could be even more probable. *Borrelia miyamotoi* has already been detected in *Ixodes* ticks collected from dogs in Germany [22], and was detected in questing *Ixodes* ticks in Spain [23,24,25]. Further studies perhaps based on experimental infections may clarify the issue of serological responses related to *B. hispanica* or *B. miyamotoi* in dogs.

The cat presented with weight loss, abdominal breathing, and a regenerative anemia, but in contrast to both canine cases, without thrombocytopenia, supporting the idea that the observed reduction in platelets in both dogs could have been the result of a co-infection. As a result of the immediate onset of doxycycline treatment, the culturing of *Borrelia* in both canine cases was not successful. As the cat blood was taken before the onset of antibiotic treatment, an in vitro cultivation of *Borrelia* was successful (Figure 1B), but only with an RF-medium (MKP basic medium supplemented with FCS). Highly mobile spirochetes were observed after 10 days of cultivation. In the BSK-H medium, a non-motile spirochete was found, while in the MKP medium and BSK-Y medium, no spirochetes were observed.

In the molecular analyses of 16S rRNA and MLST, PCR products were obtained for all three samples. For dog 1 and the cat, sequences for several housekeeping loci were obtained, while for dog 2, only the 16S rRNA locus was successfully amplified. To obtain initial information on the species designation of the isolates, a BLAST search was conducted using 16S rRNA sequences. In this search, the highest similarity scores (100 %, Table 3) were received for *B. hispanica*. For four housekeeping loci, *clpX, pepX, pyrG,* and *recG*, sequences of good quality were obtained for the cat isolate, while for dog 1, three loci produced good sequence data (*clpX, pyrG,* and *rplB*). A comparison of these sequences with the available data in GenBank showed that the highest BLAST scores (97% or 98%) were in all cases for *B. crocidurae*, *B. duttonii,* and *B. recurrentis,* while the BLAST similarity scores dropped to much lower values for *B. persica* (Table 3). A search in the MLST database at www.pubmlst.org/borrelia revealed that all of the MLST housekeeping sequences showed closest matches to *B. hispanica*—*clpX* was identified to be allele 210 and *rplB* allele 244, both representing *B. hispanica*. The closest matches (meaning the sequences were not identical, but differed in some bases) were found for *pepX* to allele 225 (three differences), for *pyrG* to allele 232 (seven and one differences in dog 1 and the cat, respectively), and for *recG* to allele 244 (three differences), further confirming that the isolates belonged to *B. hispanica*.

**Figure 2:**
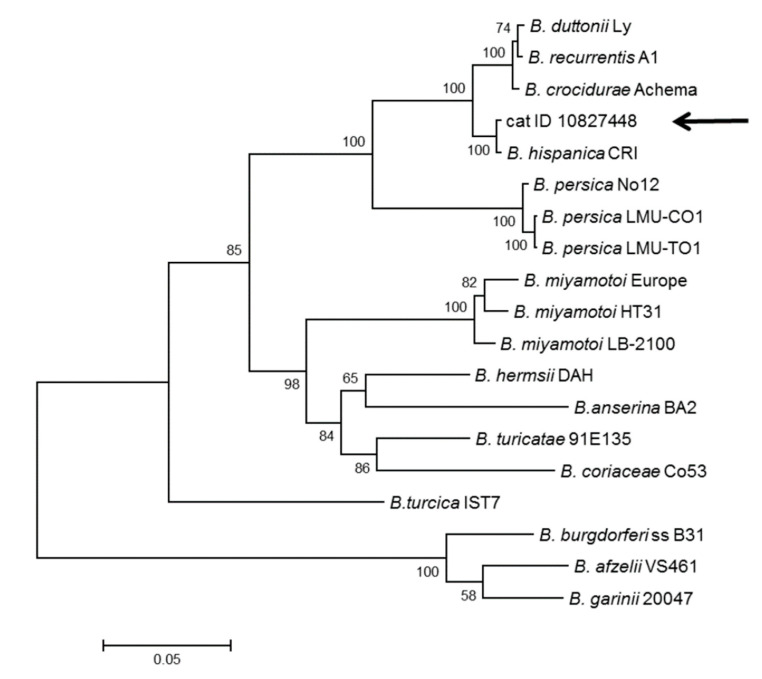
Molecular phylogenetic analysis by the maximum likelihood method based on the sequences of four housekeeping loci (*clpX, pepX, pyrG*, and *recG*). The tree with the highest log likelihood (−13,651.6897) is shown. The bootstrap value (percentage of trees in which the associated taxa clustered together) is shown next to the branches. The tree is drawn to scale; scale bar = number of substitutions per site. The analysis involved 19 nucleotide sequences. There were a total of 2443 positions in the final dataset.

**Figure 3:**
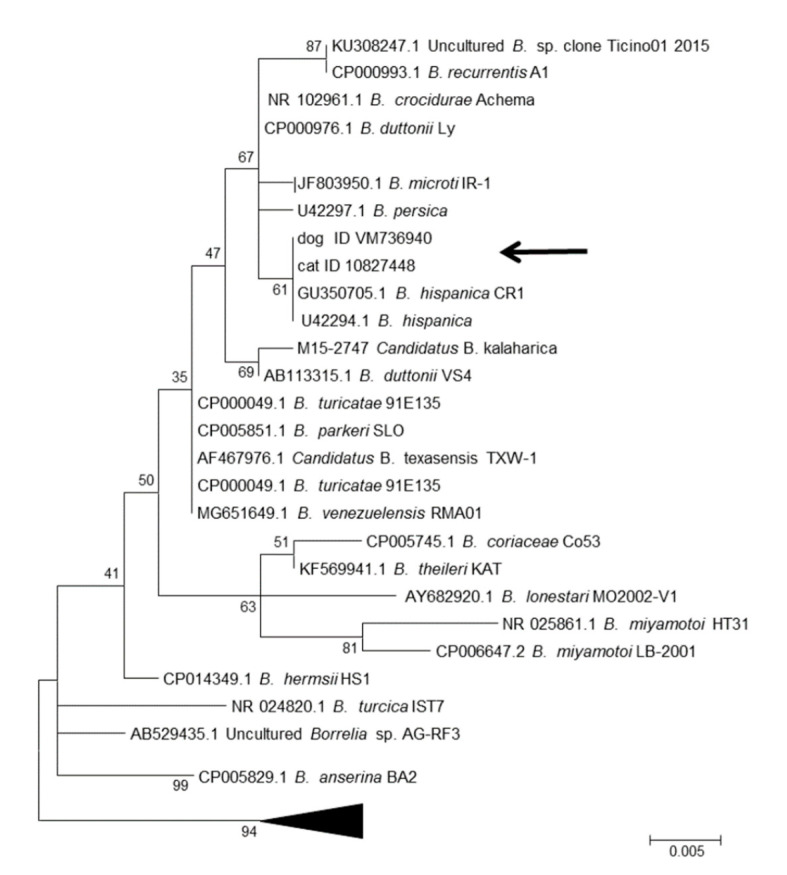
Molecular phylogenetic analysis by maximum likelihood method 16S rRNA. The tree with the highest log likelihood (−1091.6698) is shown. The bootstrap value (percentage of trees in which the associated taxa are clustered together) is shown next to the branches. The tree is drawn to scale; scale bar = number of substitutions per site. The analysis involved 43 nucleotide sequences, and the GenBank accession number, species names, and isolate names are given. There were a total of 418 positions in the final dataset. The subtree containing 17 *B. burgdorferi* sensu lato genospecies is collapsed for clarity.

The phylogenetic analyses of 16S rRNA and the concatenated sequences of four housekeeping loci from the cat isolate showed that the isolates formed a sister clade to *B. hispanica* (Figure 2 and Figure 3).

## 5. Conclusions

This is the first report of clinical cases caused by the relapsing fever spirochete *B. hispanica* in dogs and cats from Europe (Spain). Some clinical signs and/or laboratory values might have been influenced by the presence of other vector-borne pathogens.

## Figures and Tables

**Table 1:** Clinical signs and clinicopathological abnormalities in *Borrelia hispanica* infected animals.

Sample/Date	Dog 1	Dog 2	Cat
Date of sampling	30 October 2014	21 June 2018	21 July 2018
Lab sample ID	VM531519/10015666	VM73694/11024511	10827448
*Clinical signs*	Lethargic	Lethargic	Cachexia
	Pale mucosa, hematocrit 30.2% (reference range 38.3–56.5%)	High body temperature (39 °C)	Abdominal respiration
*Clinico-pathological abnormalities*	Non-regenerative anemia; (erythrocytes 3.98 m/µL, hemoglobin 8.9 g/dL, hematocrit 29.4%, reticulocytes 58,108/µL)	Leukocytosis (20.3 g/L)	Regenerative anemia (low erythrocytes 2.45 m/µL, hemoglobin 3.3 g/dL, and hematocrit 12.9%; hematocrit reference range 25–45%)
	Thrombocytopenia (23 tsd.)	Thrombocytopenia (92 tsd.)	High reticulocytes numbers (139,895/µL); mild monocytosis
*Blood taken*	February 2014 March 2014 October 2014	June 2018	July 2018
*Blood smear*	*Borrelia* positive (second presentation)	*Borrelia* positive (very few bacteria) *Babesia* spp. positive (low level)	*Borrelia* positive
*Previous treatment*	Doxycycline (2 courses; Feb 2014 and March 2014)	None	None

**Table 2:** Primer * used to amplify MLST housekeeping genes in *Borrelia hispanica.*

Locus	Primer Name	Primer Sequence
*clpA*	clpAF1258	5′-GATAAAGCTTTTGA**YY**TATTAGATGG-3
*clpA*	clpAR2276	5′-TCATATTT**D**AT**R**GTDTCGTC-3′
*clpX*	clpXF104	5′-CTGTTGC**Y**ATTTGTTTTGAATG**Y**TC-3′
*clpX*	clpXR1277	5′-TAAAGTTCTTTTGCCCAAGG-3′
*pepX*	pepF361	5′-AGAGA**Y**TTAAGYTTA**K**CAGG-3′
*pepX*	pepR1207	5′-C**Y**ATAGTTTCTCTTAAAGA**Y**TGC-3
*pyrG*	pyrF379	5′-TATTTAGG**K**AGAACTGTACAGC-3
*pyrG*	pyrR1375	5′-CAAGTCGCATTGT**W**GCAC-3
*recG*	recF898	5′-GC**K**TTTCT**M**TCTAG**Y**ATTCC-3
*recG*	recR1779	5′-TTC**R**GTTAAAGGTTCCTTATAAAG-3
*rplB*	rplF3	5′-GGAGAAAAATATGGG**K**ATTAAGAC-3
*rplB*	rplR769	5′-G**R**CCCCAAGG**W**GATAC-3
*uvrA*	uvrF1170	5′-GAGGCGTTATCTT**W**CAAC-3
*uvrA*	uvrR2181	5′-AGACTCTGGAAGCTT**W**GC-3

* Degenerate bases are shown in boldface letters.

**Table 3:** Allele numbers, GenBank accession numbers, and Basic Local Alignment Search Tool (BLAST) hits in GenBank.

Locus/Sample	Species and Isolate	Coverage	Similarity	GenBank Accession Number
*clpX*/dog1 (#allele 210)/cat (#allele 210)	*Borrelia crocidurae* str. Achema	100%	97%	CP003426.1
	*Borrelia recurrentis* A1	100%	97%	CP000993.1
	*Borrelia duttonii* Ly	100%	97%	CP000976.1
	*Borrelia crocidurae* DOU	100%	96%	CP004267.1
	*Borrelia persica* strain LMU-T01	99%	91%	KP826804.
*pepX*/cat (#allele 263)	*Borrelia recurrentis* A1	100%	98%	CP000993.1
	*Borrelia crocidurae* DOU	100%	97%	CP004267.1
	*Borrelia crocidurae* str. Achema	100%	97%	CP003426.1
	*Borrelia duttonii* Ly	99%	97%	CP000976.1
	*Borrelia persica* strain LMU-T01	100%	90%	KP826805.1
*pyrG*/dog1 (#allele 274)	*Borrelia crocidurae* str. Achema	100%	98%	CP003426.1
	*Borrelia recurrentis* A1	100%	97%	CP000993.1
	*Borrelia duttonii* Ly	100%	97%	CP000976.1
	*Borrelia persica* strain LMU-T01	100%	89%	KP826806.1
*pyrG*/cat (#allele 273)	*Borrelia crocidurae* DOU	100%	98%	CP004267.1
	*Borrelia crocidurae* str. Achema	100%	98%	CP003426.1
	*Borrelia recurrentis* A1	100%	98%	CP000993.1
	*Borrelia duttonii* Ly	100%	98%	CP000976.1
	*Borrelia persica* strain LMU-T01	100%	90%	KP826806.1
*recG*/cat (#allele 288)	*Borrelia crocidurae* str. Achema	100%	97%	CP003426.1
	*Borrelia crocidurae* DOU	100%	97%	CP004267.1
	*Borrelia duttonii* Ly	100%	97%	CP000976.1
	*Borrelia recurrentis* A1	100%	96%	CP000993.1
	*Borrelia persica* strain LMU-T01	99%	89%	KP826807.1
*rplB*/dog1 (#allele 206)	*Borrelia crocidurae* str. Achema	100%	98%	CP003426.1
	*Borrelia recurrentis* A1	100%	98%	CP000993.1
	*Borrelia duttonii* Ly	100%	98%	CP000976.1
	*Borrelia crocidurae* DOU	100%	98%	CP004267.1
	*Borrelia persica* strain LMU-T01	98%	92%	KP826807.1
16S rRNA/dog2 (* MN175320)/cat (* MN173954)	*Borrelia hispanica* strain CR1	100%	100%	GU350705.1
	*Borrelia hispanica* 16S rRNA gene	100%	100%	DQ057988.1
	*Borrelia crocidurae* str. Achema	100%	99%	CP003426.1
	*Borrelia hispanica* strain Sp3	100%	99%	GU350706.1
	*Borrelia crocidurae* strain 7-10TO58	100%	99%	GQ358198.1

# allele numbers and isolate information are available at the *Borrelia* MLST database pubmlst.org/borrelia/; * GenBank accession number.
